# The incidence of smoking and risk factors for smoking initiation in medical faculty students: cohort study

**DOI:** 10.1186/1471-2458-6-128

**Published:** 2006-05-10

**Authors:** Yesim Senol, Levent Donmez, Mehtap Turkay, Mehmet Aktekin

**Affiliations:** 1 Specialist in Public Health, Assistant Professor, Department of Medical Education, Akdeniz University Medical Faculty, Antalya, Turkey; 2 Associate Professor, Department of Public Health, Akdeniz University Medical Faculty, Antalya, Turkey; 3 Specialist in Public Health, Department of Medical Education, Akdeniz University Medical Faculty, Antalya, Turkey; 4 Professor, Department of Public Health & Medical Education, Akdeniz University Medical Faculty, Antalya, Turkey

## Abstract

**Background:**

Medical education requires detailed investigation because it is a period during which the attitudes and behaviors of physicians develop. The purpose of this study was to calculate the yearly smoking prevalence and incidence rates of medical faculty students and to identify the risk factors for adopting smoking behaviour.

**Methods:**

This is a cohort study in which every student was asked about their smoking habits at the time of first registration to the medical faculty, and was monitored every year. Smoking prevalence, yearly incidence of initiation of smoking and average years of smoking were calculated in analysis.

**Results:**

At the time of registration, 21.8% of the students smoked. At the end of six years, males had smoked for an average of 2.6 ± 3.0 years and females for 1.0 ± 1.8 years (p < 0.05). Of the 93 medical students who were not smokers at the time of registration, 30 (32.3%) were smokers at the end of the 6 years of the course.

**Conclusion:**

The first 3 years of medical education are the most risky period for initiation of smoking. We found that factors such as being male, having a smoking friend in the same environment and having a high trait anxiety score were related to the initiation of smoking. Targeted smoking training should be mandatory for students in the Medical Faculty.

## Background

Smoking is a major public health problem all around the world, especially in developing countries. It is estimated that by 2025, 75% of early deaths in developing countries will be due to smoking-related illnesses [1]. One third of the global population aged 15 years and over (approximately 1.1 billion people) are current smokers. Most of them live in developing countries (800 million) and most are males (700 million) [2]. In Turkey, 44% of adults are smokers (60–65% in males, 20–24% in females) [2]. Among European countries, Turkey is second only to Greece in cigarette consumption per person. The percentage of smokers is steadily increasing in Turkey, in contrast to developed countries [2].

Physicians have an important role in the struggle against smoking. Besides dealing with smoking-related health problems, they are also expected to represent a role model for the population by adopting a non-smoking lifestyle [3]. Their dedicated opposition to smoking may have a huge effect on society [4,5]. However, current evidence shows that most physicians in Turkey smoke [6,7] and would probably not influence the population positively in this respect. The time during medical education seems to be the ideal period to establish such a positive influence. In order to assess the importance of this period, many investigators have studied the smoking behavior of medical students [8-12]. However, most of these studies have been cross-sectional and do not suffice to elucidate the adoption of smoking behaviour. In our opinion, it is important to define the characteristics of students who started to smoke during medical education in order to identify high-risk groups and to plan effective preventative strategies. A subsequent prospective follow-up study, allowing smoking incidence to be calculated for each year of medical education, would be useful in determining the riskiest period for initiation of smoking.

The purpose of this study was to investigate the risk factors for initiation of smoking among medical students and to determine the annual prevalence and incidence rates of smoking.

## Methods

This cohort study was carried out among students of Akdeniz University Faculty of Medicine, established 30 years ago.

### Design of the study

**Figure 1:**
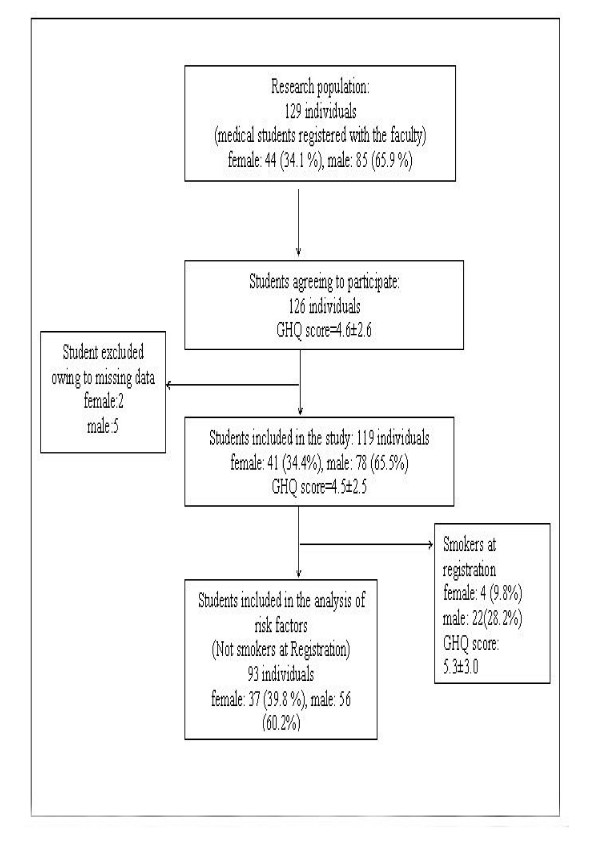
Design of the study.

The study cohort consisted of 126 (97.6%) of the 129 students registered at Akdeniz University Medical Faculty in 1996 who agreed to participate in the study. The authors prepared a questionnaire that included questions about smoking status, age at which smoking started, other smokers among family members or friends, alcohol intake, tea and coffee consumption, location of students' family (province/county), nutritional status, existence of a boy/girl friend and mental health. All participants were asked to complete the questionnaire at the date of first registration and at the beginning of each academic year throughout the 6 years of medical education. To ensure the anonymity of subjects, the students were monitored by code numbers given at the time of the first interview. The code numbers were issued by a person who was not involved the study. Students used these code numbers in the questionnaires instead of their names. Ethical approval to conduct this study was granted by Akdeniz University Faculty of Medicine Ethics Committee and written informed consent was obtained from each student. In order to avoid possible confounding effects of exams and/or other school-related problems, the students were asked to complete the questionnaires during the first month of the first semester each year. Seven students were excluded from the study during the follow-up period because they did not complete the questionnaire correctly. The study was continued with the remaining 119 students. The characteristics of initiation of smoking were determined, and the incidence of initiation was calculated for the 93 participants who were not smokers at time of the first registration (Figure 1).

### Variables

Students smoking ≤ 1 cigarettes per day were considered regular smokers [13]. Any student who had had ≤ 1 alcoholic drink per week during the past year was considered an alcohol taker, while those who had not, or had drunk only on special occasions over the same period, were classified as alcohol non-takers. The students were asked to rate their nutritional status as sufficient if they had breakfast regularly and consumed vegetables, fruit and meat adequately. Students who took part in physical exercise for more than 20 mins at least twice per week were accepted as exercisers. The participants were also asked to complete the General Health Questionnaire-12 (GHQ), Spielberger State-Trait Anxiety Inventory (STAI) 1–2 and Beck Depression Inventory (BDI) to determine their mental health status [14-16].

The 12- item GHQ was chosen because of its well-established validity in student samples [17]. Each item in the GHQ has a standard 0-0-1-1 scoring method (maximum score of 12). Depression was assessed using the BDI, a 21-item self-reporting questionnaire in which each item is scored zero to three [16]. Higher scores indicate a higher level of depression. The total STAI score was obtained to determine anxiety. The STAI has a 20-item form for state anxiety and a 20-item form for trait anxiety and is scored from one to four [15]. Each form allows a minimum score of 20 and a maximum score of 80.

### Analyses

Smoking prevalence, yearly incidence of initiation of smoking and average years of smoking were calculated.

#### Incidence calculation

Incidence was defined as the number of new cases occurring during a given time interval divided by the population at risk at the beginning of that interval [18]. In accordance with this definition, the incidence was calculated as follows:

Incidence=The number of students who began smoking during the relevant yearThe number of non−smoking students at the beginning of the relevant year
 MathType@MTEF@5@5@+=feaafiart1ev1aaatCvAUfKttLearuWrP9MDH5MBPbIqV92AaeXatLxBI9gBaebbnrfifHhDYfgasaacH8akY=wiFfYdH8Gipec8Eeeu0xXdbba9frFj0=OqFfea0dXdd9vqai=hGuQ8kuc9pgc9s8qqaq=dirpe0xb9q8qiLsFr0=vr0=vr0dc8meaabaqaciaacaGaaeqabaqabeGadaaakeaacqWGjbqscqWGUbGBcqWGJbWycqWGPbqAcqWGKbazcqWGLbqzcqWGUbGBcqWGJbWycqWGLbqzcqGH9aqpdaWcaaqaaiabdsfaujabdIgaOjabdwgaLjabbccaGiabd6gaUjabdwha1jabd2gaTjabdkgaIjabdwgaLjabdkhaYjabbccaGiabd+gaVjabdAgaMjabbccaGiabdohaZjabdsha0jabdwha1jabdsgaKjabdwgaLjabd6gaUjabdsha0jabdohaZjabbccaGiabdEha3jabdIgaOjabd+gaVjabbccaGiabdkgaIjabdwgaLjabdEgaNjabdggaHjabd6gaUjabbccaGiabdohaZjabd2gaTjabd+gaVjabdUgaRjabdMgaPjabd6gaUjabdEgaNjabbccaGiabdsgaKjabdwha1jabdkhaYjabdMgaPjabd6gaUjabdEgaNjabbccaGiabdsha0jabdIgaOjabdwgaLjabbccaGiabdkhaYjabdwgaLjabdYgaSjabdwgaLjabdAha2jabdggaHjabd6gaUjabdsha0jabbccaGiabdMha5jabdwgaLjabdggaHjabdkhaYbqaaiabdsfaujabdIgaOjabdwgaLjabbccaGiabd6gaUjabdwha1jabd2gaTjabdkgaIjabdwgaLjabdkhaYjabbccaGiabd+gaVjabdAgaMjabbccaGiabd6gaUjabd+gaVjabd6gaUjabgkHiTiabdohaZjabd2gaTjabd+gaVjabdUgaRjabdMgaPjabd6gaUjabdEgaNjabbccaGiabdohaZjabdsha0jabdwha1jabdsgaKjabdwgaLjabd6gaUjabdsha0jabdohaZjabbccaGiabdggaHjabdsha0jabbccaGiabdsha0jabdIgaOjabdwgaLjabbccaGiabdkgaIjabdwgaLjabdEgaNjabdMgaPjabd6gaUjabd6gaUjabdMgaPjabd6gaUjabdEgaNjabbccaGiabd+gaVjabdAgaMjabbccaGiabdsha0jabdIgaOjabdwgaLjabbccaGiabdkhaYjabdwgaLjabdYgaSjabdwgaLjabdAha2jabdggaHjabd6gaUjabdsha0jabbccaGiabdMha5jabdwgaLjabdggaHjabdkhaYbaaaaa@E8E8@

We calculated yearly incidence rates (for each year) as well as total incidence (for the entire 6 years). Any student included in the calculation of incidence of initiation in a particular year was excluded from calculations of incidence in the following years.

#### Yearly prevalence

Prevalence is defined as the number of individuals with a given behaviour at a given moment divided by the population at risk at that moment [18].

Yearly prevalence=The number of students who smoked at the time of completion of the questionnaireThe number of non−smoking students at the beginning of the relevant year
 MathType@MTEF@5@5@+=feaafiart1ev1aaatCvAUfKttLearuWrP9MDH5MBPbIqV92AaeXatLxBI9gBaebbnrfifHhDYfgasaacH8akY=wiFfYdH8Gipec8Eeeu0xXdbba9frFj0=OqFfea0dXdd9vqai=hGuQ8kuc9pgc9s8qqaq=dirpe0xb9q8qiLsFr0=vr0=vr0dc8meaabaqaciaacaGaaeqabaqabeGadaaakeaacqWGzbqwcqWGLbqzcqWGHbqycqWGYbGCcqWGSbaBcqWG5bqEcqqGGaaicqWGWbaCcqWGYbGCcqWGLbqzcqWG2bGDcqWGHbqycqWGSbaBcqWGLbqzcqWGUbGBcqWGJbWycqWGLbqzcqGH9aqpdaWcaaqaaiabdsfaujabdIgaOjabdwgaLjabbccaGiabd6gaUjabdwha1jabd2gaTjabdkgaIjabdwgaLjabdkhaYjabbccaGiabd+gaVjabdAgaMjabbccaGiabdohaZjabdsha0jabdwha1jabdsgaKjabdwgaLjabd6gaUjabdsha0jabdohaZjabbccaGiabdEha3jabdIgaOjabd+gaVjabbccaGiabdohaZjabd2gaTjabd+gaVjabdUgaRjabdwgaLjabdsgaKjabbccaGiabdggaHjabdsha0jabbccaGiabdsha0jabdIgaOjabdwgaLjabbccaGiabdsha0jabdMgaPjabd2gaTjabdwgaLjabbccaGiabd+gaVjabdAgaMjabbccaGiabdogaJjabd+gaVjabd2gaTjabdchaWjabdYgaSjabdwgaLjabdsha0jabdMgaPjabd+gaVjabd6gaUjabbccaGiabd+gaVjabdAgaMjabbccaGiabdsha0jabdIgaOjabdwgaLjabbccaGiabdghaXjabdwha1jabdwgaLjabdohaZjabdsha0jabdMgaPjabd+gaVjabd6gaUjabd6gaUjabdggaHjabdMgaPjabdkhaYjabdwgaLbqaaiabdsfaujabdIgaOjabdwgaLjabbccaGiabd6gaUjabdwha1jabd2gaTjabdkgaIjabdwgaLjabdkhaYjabbccaGiabd+gaVjabdAgaMjabbccaGiabd6gaUjabd+gaVjabd6gaUjabgkHiTiabdohaZjabd2gaTjabd+gaVjabdUgaRjabdMgaPjabd6gaUjabdEgaNjabbccaGiabdohaZjabdsha0jabdwha1jabdsgaKjabdwgaLjabd6gaUjabdsha0jabdohaZjabbccaGiabdggaHjabdsha0jabbccaGiabdsha0jabdIgaOjabdwgaLjabbccaGiabdkgaIjabdwgaLjabdEgaNjabdMgaPjabd6gaUjabd6gaUjabdMgaPjabd6gaUjabdEgaNjabbccaGiabd+gaVjabdAgaMjabbccaGiabdsha0jabdIgaOjabdwgaLjabbccaGiabdkhaYjabdwgaLjabdYgaSjabdwgaLjabdAha2jabdggaHjabd6gaUjabdsha0jabbccaGiabdMha5jabdwgaLjabdggaHjabdkhaYbaaaaa@06A7@

In addition, *Total prevalence *was the number of students who smoked at least once during the 6 years of medical education divided by the total student population.

#### Average years of smoking

In order to calculate this variable we added the smoking duration of each student and divided this number by the whole study population. Years of smoking before the entering the medical faculty were included in this calculation.

In order to investigate the relationship between mental health status and smoking behavior, the six-year mean scores on the three different scales were compared between those who started smoking and those of non-smokers.

Statistical analyses of the variables related to initation of smoking involved only the 93 students who were non-smokers at initial registration. Chi-square tests, Fisher's exact chi-square tests and logistic regression analyses were used for statistical evaluations. To determine the relative importance of each predictor variable on the initiation of smoking, multivariate analysis was performed by logistic regression. The Forward Conditional model was used. Mental Health Scales were entered in the model as "continuous" variables. Gender, family location, smokers among friends, smoking family members, tea consumption, alcohol use, coffee consumption, boyfriend/girlfriend, sports and nutrition were entered in the model as "categorical" variables. In addition, a replacement-of-missing-value operation was undertaken for the 7 students whose data showed deficits in some years, without affecting the data distribution or mean values. The SPSS "replace" function was used to replace the missing values by the means of all values. The order of the subjects was randomized. The replacement operation was performed only for regression analysis. Statistical significance was accepted when p ≤ 0.05.

## Results

Of the total 119 medical students included in the study, 78 (65.5%) were male and 41 (34.5%) were female. A total of 26 (21.8%) students were already smokers at the time of registration.

At the end of six years, the smoking duration in males (2.6 ± 3.0 years) was significantly higher than that in females (1.0 ± 1.8 years) (p < 0.05), but the age at which smoking began showed no significant difference between the genders (for males 17.0 ± 2.6 years; for females 17.7 ± 1.7 years).

The annual smoking prevalence and incidence of new starters are shown in Table 1. The riskiest period of medical education for initiation of smoking is the first 3 years. Among the 93 non-smokers at the time of registration, 12.5% (95% CI 7.5–21.2) started smoking at the end of the first year, 7.4% (95% CI 3.5–15.2) at the end of the second year and 9.3% (95% CI 4.6–18.1) at the end of the third year. Altogether, 30 of these original non-smokers (32.3%) were smokers by the end of the 6 years. A total of 56 students (47.1%) tried smoking at least once or became smokers during the 6 year period.

The characteristics of the initial non-smokers who started smoking during medical education are shown in Tables 2 and 3. The unadjusted risk of initiation was increased 2.03 times by having smokers among friends, and 1.82 times by inadequate nutrition (p < 0.05 for both) (Table 2). The trait anxiety score was significantly higher in these students (45.6 ± 6.0 points) than in those who did not start smoking (42.2 ± 7.3 points) (p < 0.05) (Table 3). No statistically significant relationships were found between initiation of smoking and the other variables investigated.

Table 4 shows the results of the logistic regression analysis performed to eliminate possible confounding relationships among the variables and indicates the variables that showed statistical significance. In this analysis, the variables in Tables 2 and 3 were described as "categorical" and "continuous", respectively. The presence of smokers among family or friends, being male and having a high trait anxiety score were the significant risk factors for initiation of smoking among medical students according to logistic regression analysis; the other variables were not significant.

## Discussion

The present study had both advantages and limitations. As a prospective study, it allowed us to calculate the incidence of smoking for each single year and thus enabled us to determine the most risky period for initiation during medical education, and the best period to implement available anti-smoking strategies. On the other hand, the exclusion of several students decreased the research population, and missing data for some years were major limitations. Fortunately, the number of students omitted from the study was relatively low (10) and their characteristics were similar to the general distribution (Figure 1). Although we included all students registered, the sample size may still not be large enough to determine the effects of all independent variables on initiation of smoking.

Tobacco use is frequent among medical students. Although the annual smoking prevalence rates found in our study were between 21.8% and 33.6% (Table 1), similar to those reported in previous studies [12,19-21], the total prevalence rate during medical education (47.1%) seems higher, possibly because of the methodological difference between total and cross-sectional prevalence calculations. The smoking rate was 22.5% in the Comenius University of Slovakia [19], 29.0% in the Zagreb Medical Faculty [20] and 31.7% in seven British Medical Faculties [22]. It was reported to be 23.6% for the first year and 27.3% for the fifth year for medical students in Australia [12]. Similar rates for students in the first (26.7%) and fifth (24.4%) classes were noted at Columbia Medical Faculty [21]. As these studies were all cross-sectional, they reflected only the rates for the year of the study. It is not possible to ascertain the exact rates for new and/or existing smokers, since some people may stop smoking. If primary prevention against initiation of smoking is taken into consideration, then it is obvious that everyone who has ever tried smoking must be considered within the risk group. Therefore, cross-sectional calculations seem to have some limitations in reflecting the actual situation.

If the present study had been designed cross-sectionally, we would have erroneously concluded that the risk was highest in the fourth year of medical school (Table 1). Actually, the highest incidence of initiation occurred in the first year, perhaps because of adaptation to a new educational atmosphere, difficulties in relationships with new friends and the intense anxiety experienced during the first year. Previous studies have shown that university life increases the stress levels of students [23,24]. Our findings, like those of previous studies [12,25,26], suggest that medical education does not prevent or reduce smoking; moreover, tobacco use increases over time.

The factors affecting cigarette consumption were defined in detail in previous studies [12,26-28]. We found that factors such as being male, presence of a smoking friend in shared accomodation (e.g. home or hostel), insufficient nutrition and having a high trait anxiety score (Tables 2, 3, 4) were significantly associated with initiation of smoking. In contrast to others [27-31], however, we found that a smoker in the family, alcohol intake and dating had no significant effects on the adoption of smoking behaviour. The smoking-family relationship emphasized in several studies was probably based on family-children interactions in the pre-university period. We excluded the smoking students at initial registration, so our findings did not reflect the effects of interactions with family prior to medical education. It is well known that friends have more influence than family members on initiation of smoking during education [28].

Smoking frequency was higher among males than females in our study. However, there was no significant difference between males and females regarding the age of onset of smoking. Also, boys showed a 2.88 times greater risk of initiation of smoking than girls. Traditional and/or cultural norms may play a role in the low starting rates observed in females [32].

In agreement with the finding that people eating a balanced diet with vegetables/fruits and having regular breakfasts are less likely to smoke, while those with an irregular eating routine are more likely [33], we found a significant relationship between nutrition and initiation of smoking. This relationship may have been affected by a third variable that we did not take into consideration.

Another major finding of the present study was the relationship between initiation of smoking and the trait anxiety score, which reflected the mental state of the student (Tables 3 and 4). Loss of self-confidence, a socially withdrawn character, a depressive personality and anxiety are frequent among smokers [34-36]. In addition, some studies have reported that smoking is perceived among young people as beneficial for improving social relations and coping with anxiety, and this perception may well encourage them to consume cigarettes [34]. It has also been suggested that mental health state and smoking may have a common genetic predisposition [36]. Despite current evidence for a relationship between mental health state and smoking, the nature of this interaction remains unexplained [36]. We found that the risk of initiation of smoking increased with the trait anxiety score. Given that medical education causes anxiety, which negatively affects the mental health of students, and that the mental health scores of students increase dramatically during the first year of this education period [37], medical education may possibly have an indirect negative effect on smoking.

Our findings suggest that any preventative anti-smoking measures must be implemented in the first year of medical education with more focus on males, since male students with high trait anxiety scores and with at least one smoking friend are at high risk of starting smoking. Both social and psychological support starting in the first class of medical education would be effective in the struggle against smoking. Precautions such as increasing cigarette prices and banning tobacco advertising, and supporting activities such as peer education on smoking, as well as offering relevant courses in the first year curriculum at medical school, may be effective for young people at high risk of adopting smoking behaviour. However, it would not be adequate to implement such programs only for faculty years. Enlargement of both scope and area of implementation would be needed to increase the success rate. Low smoking rates in both the general and student populations in countries with rigid preventative methods [3,38] prove that effective outcomes may be obtained with the aforementioned interventions.

However, it should be kept in mind that childhood through adolescence is a very important age for development of attitues towards smoking, so preventative measures should be implemented as early as elementary school years. Education in medical faculties only partially influences attitudes. In medical education, greater attention should be paid to the treatment of smoking dependence and ways of preventing smoking in order to prepare the students, as future health prefessionals, for their role in prevention.

## Conclusion

As a in conclusion, the first 3 years of medical education are the most risky period for initiation of smoking. Male gender, presence of a smoking friend in shared accomodation, insufficient nutrition and having a high trait anxiety score were the significant factors on smoking initiation in our study. Interventions including both social and psychological support starting in the early years of medical education are needed.

## Competing interests

The author(s) declare that they have no competing interests.

## Authors' contributions

YS was responsible for design, acquisition of data, analysis, interpretation of data and revised the manuscript. LD was responsible for analysis and interpretation of data. MT was responsible for acquisition of data and final approval. MA was responsible for the design and final approval. All authors read and approved the final manuscript.

## Pre-publication history

The pre-publication history for this paper can be accessed here:



## Figures and Tables

**Table 1:** Annual smoking frequency of students and percentage of new starters.

	**Prevalence**			Incidence*		
Years	n	Smokers	% (%95 CI)	n	New smokers	% (%95 CI)
1^st ^(register)	119	26	21.8 (15.3–30.1)	-	-	-
2^nd ^	119	35	29.9 (21.9–38.2)	93	12	12.9 (7.5–21.2)
3^rd ^	119	38	31.9 (24.2–40.8)	81	6	7.4 (3.5–15.2)
4^th ^	119	40	33.6 (24.2–40.8)	75	7	9.3 (4.6–18.1)
5^th ^	112	29	25.9 (18.7–34.7)	68	2	2.9 (1.0–10.1)
6^th ^	115	31	27.0 (19.7–35–7)	66	3	4.5 (1.6–12.5)

*: For incidence calculations, only the new smokers within a particular year were taken into account; e.g. a group of students who smoked in the first year (21.8%) was not included in the calculations for the second year (29.9%).

**Table 2:** Variables affecting initiation of smoking (new smokers) during 6 years of medical education.

**Variables**	**n**	**New smokers**	**%**	**Relative risk**	**95% CI**	p*
Gender						

Female	37	8	21.6			
Male	56	22	39.3	1.817	(0.91–3.64)	0.074

Family location						

Urban	59	17	28.8			
Rural	34	13	38.2	1.327	(0.74–2.38)	0.349

Other smokers among friends						

No	65	16	24.6			
Yes	28	14	50.0	2.031	(1.16–3.57)	0.016

Smoking family members						

No	39	10	25.6			
Yes	54	20	37.0	1.444	(0.76–2.73)	0.246

Tea consumption						

No	6	3	50.0			
Yes	87	27	31.0	0.621	(0.26–1.47)	0.383**

Alcohol use						

No	84	27	32.1			
Yes	9	3	33.3	1.037	(0.39–2.75)	1.0**

Coffee consumption						

No	40	10	25.0			
Yes	53	20	37.7	1.509	(0.80–2.86)	0.193

Boyfriend/girlfriend						

No	59	16	27.1			
Yes	34	14	41.2	1.518	(0.85–2.71)	0.163

Sports						

Playing	13	4	30.8			
Not playing	80	26	32.5	1.056	(0.44–2.53)	1.0**

Nutrition						

Sufficient	73	20	27.4			
Insufficient	20	10	50.0	1.825	(1.03–3.25)	0.05

*: Chi-square test, **: Fisher's exact test, %: individuals who started smoking during 6 years/total number of individuals, CI: confidence interval.

**Table 3:** Mental health scales of students who started or did not start smoking during the 6 years of medical education.

**Mental State Scale**	**Started smoking (n = 30) Mean ± SD**	**Not started smoking (n = 63) Mean ± SD**	p*
STAI-State	41.9 ± 7.1	38.8 ± 8.2	0.078
STAI-Trait	45.7 ± 6.0	42.2 ± 7.3	0.027
BDI	9.8 ± 5.6	7.5 ± 5.7	0.078
GHQ	4.6 ± 2.2	4.2 ± 2.4	0.480

*: Student t test.

*STAI_State: *Spielberger State-Trait Anxiety Inventory

*STAI- Trait*: Spielberger State-Trait Anxiety Inventory

*BDI: *Beck Depression Inventory

*GHQ: *General Health Questionnaire-12

**Table 4:** Odds ratios (OR) for the factors affecting initiation of smoking among medical students.

**Variable**	**OR**	**95% CI**	***p***
Being male	2.88	1.02–8.14)	0.045
Other smokers among friends	3.54	1.30–9.66	0.014
Trait anxiety score	1.09	1.02–1.18	0.017

p: probability, CI: confidence interval

## References

[B1] WHO (1998). Guidelines for controlling and monitoring the tobacco epidemic.

[B2] WHO (1997). Tobacco or health A global status report.

[B3] Mangus SR, Hawkins CE, Miller MJ (1998). Tobacco and alcohol use 1996 medical School Graduates. JAMA.

[B4] Pederson L (1982). Compliance with pshysician advice to quit smoking: a review of the literature. Prev Med.

[B5] Jamrozik K, Vessey M, Fowler G, Wald N, Parker G, Van Vunakis H (1984). Controlled trial of three different anti- smoking interventions in general practice. BMJ.

[B6] Dedeoglu N, Donmez L, Aktekin M (1994). Tobacco use among health personnel in Antalya. Turkish Journal of Smoking and Health.

[B7] Bilir N, Dogan B, Yildiz AN (1997). Smoking behaviour and attitudes (Ankara- Turkey).

[B8] Mas A, Nerin I, Burrueco M, Cordero J, Guillen D, Jimenez-Ruiz J, Sobradillo V (2004). Smoking habits among sixth-year medical students in Spain. Arch Bronconeumol.

[B9] Brenner H, Scharrer SB (1996). Parental smoking and sociodemographic factors related to smoking among German medical students. Eur J Epidemiol.

[B10] Hussain SF, Moid I, Khan JA (1995). Attitudes of Asian medical students towards smoking. Thorax.

[B11] Kocabas A, Burgut R, Bozdemir N (1994). Smoking patterns at different medical schools in Turkey. Tobacco Control.

[B12] Richmond RL, Kehoe L (1997). Smoking behaviour and attitudes among Australian medical students. Medical Education.

[B13] (1983). Guidelines for the conduct of tobacco smoking survey of the general population.

[B14] Goldberg DP (1972). The detection of psychiatric illness by questionnaire.

[B15] Spielberger CD, Goursuch RL, Lushene RE (1970). Manual for the State Trait Anxiety Inventory.

[B16] Beck AT (1967). Depression, Clinical, Experimental and Theoretical Aspects.

[B17] Radanovic Z, Eric LJ (1983). Validity of the General health Questionnaire in Yugoslav student population. Psychol Med.

[B18] Dawson-Saunders B, Trapp RG (1990). Basic and Clinical Biostatistics.

[B19] Baska T, Straka S, Mad'ar R (2000). Smoking habits in university students in Slovakia. Cent Eur J Public Health.

[B20] Trkuljia V, Zivcec Z, Cuk M, Lackovic Z (2003). Use of Psychoactive substance among Zagreb University Medical Students: follow-up Study. Croatian Medical Journal.

[B21] Rosselli D, Rey O, Calderon C, Rodriguez MN (2001). Smoking in Colombian Medical Schools: The hidden curriculum. Preventive Medicine.

[B22] Webb E, Ashton CH, Kelly P, Kamali F (1998). An update on British medical students' lifestyles. Medical Education.

[B23] Webb E, Ashton CH, Kelly P, Kamali F (1996). Alcohol and drug use in UK university students. Lancet.

[B24] Guthrie E, Black D, Bagalkote H, Shaw C, Campbell M, Creed F (1998). Psychological stress and burnout in medical students: a five-year prospective longitudinal study. J R Soc Med.

[B25] Vakeflliu Y, Argjiri D, Peposhi I, Agron S, Melani AS (2002). Tobacco smoking habits. Beliefs and attitudes among medical students in Trina, Albania. Preventive Medicine.

[B26] Akvardar Y, Demiral Y, Ergor G, Ergor A, Bilici M, Özer OA (2003). Substance use in a sample of Turkish medical students. Drug and Alcohol Dependence.

[B27] Morello P, Duggan A, Adger H, Anthony JC, Joffe A (2001). Tobacco Use among high school students in Buenos Aires, Argentina. American Journal of Public Health.

[B28] Azevedo A, Machado AP, Barros H (1999). Tobacco smoking among Portuguese high-school students. Bulletin of the World Health Organization.

[B29] Vitaro F, Wanner B, Brendgen M, Gosselin C, Gendreau PL (2004). Differential contribution of parents and friends to smoking trajectories during adolescence. Addictive Behaviors.

[B30] Anderson K, World Health Organization (1995). Tobacco use by young peopleI. Young people and alcohol drugs and tobacco.

[B31] Charlton AQ, Blair V (1989). Predicting the onset of smoking in boys and girls. Social Science and Medicine.

[B32] Bush J, White M, Kai J, Rankin J, Bhopal R (2003). Understanding influences on smoking in Bangladeshi and Pakistani adults: community based qualitative study. BMJ.

[B33] Kvaavik E, Meyer HE, Tverdal A (2004). Food habits, physical activity and body mass index in relation to smoking status in 40–42 year old Norwegian women and men. Preventive Medicine.

[B34] Patton GC, Hibbert M, Roiser MJ, Carlin JB, Caust J, Bowes G (1996). Is smoking associated with depression and anxiety in teenagers?. American Journal of Public Health.

[B35] Anda RF, Williamson DF, Escobedo LG, Mast EE, Giovino GA, Remington PL (1990). Depression and the dynamics of smoking. A national perspective. JAMA.

[B36] Vogel JS, Hurford DP, Smith JV, Cole AK (2003). The relationship between depression and smoking in adolescents. Adolescence.

[B37] Aktekin M, Karaman T, Senol YY, Erdem S, Erengin H, Akaydýn M (2001). Anxiety. depression and stressful life events among medical students: a prospective study in Antalya, Turkey. Medical Education.

[B38] Ringel JS, Wasserman J, Andreyeva T (2005). Effects of public policy on adolescents' cigar use: evidence from the National Youth Tobacco Survey. American Journal of Public Health.

